# *STAY-GREEN* Accelerates Chlorophyll Degradation in *Magnolia sinostellata* under the Condition of Light Deficiency

**DOI:** 10.3390/ijms24108510

**Published:** 2023-05-09

**Authors:** Mingjie Ren, Jingjing Ma, Danying Lu, Chao Wu, Senyu Zhu, Xiaojun Chen, Yufeng Wu, Yamei Shen

**Affiliations:** Zhejiang Provincial Key Laboratory of Germplasm Innovation and Utilization for Garden Plants, College of Landscape and Architecture, Zhejiang Agriculture and Forestry University, Hangzhou 311300, China

**Keywords:** *Magnolia sinostellata*, *STAY-GREEN* gene (*SGR*), chlorophyll, light deficiency, transcriptome, yeast two-hybrids analysis

## Abstract

Species of the Magnoliaceae family are valued for their ornamental qualities and are widely used in landscaping worldwide. However, many of these species are endangered in their natural environments, often due to being overshadowed by overstory canopies. The molecular mechanisms of *Magnolia*’s sensitivity to shade have remained hitherto obscure. Our study sheds light on this conundrum by identifying critical genes involved in governing the plant’s response to a light deficiency (LD) environment. In response to LD stress, *Magnolia sinostellata* leaves were endowed with a drastic dwindling in chlorophyll content, which was concomitant to the downregulation of the chlorophyll biosynthesis pathway and upregulation in the chlorophyll degradation pathway. The *STAY-GREEN* (*MsSGR*) gene was one of the most up-regulated genes, which was specifically localized in chloroplasts, and its overexpression in Arabidopsis and tobacco accelerated chlorophyll degradation. Sequence analysis of the *MsSGR* promoter revealed that it contains multiple phytohormone-responsive and light-responsive cis-acting elements and was activated by LD stress. A yeast two-hybrid analysis resulted in the identification of 24 proteins that putatively interact with MsSGR, among which eight were chloroplast-localized proteins that were significantly responsive to LD. Our findings demonstrate that light deficiency increases the expression of *MsSGR*, which in turn regulates chlorophyll degradation and interacts with multiple proteins to form a molecular cascade. Overall, our work has uncovered the mechanism by which *MsSGR* mediates chlorophyll degradation under LD stress conditions, providing insight into the molecular interactions network of MsSGR and contributing to a theoretical framework for understanding the endangerment of wild Magnoliaceae species.

## 1. Introduction

Magnoliaceae species are cultivated globally due to their high ornamental and commercial values. Unfortunately, many Magnoliaceae species are on the verge of extinction due to community succession and environmental threats to their habitats [1]. Some endangered Magnoliaceae species, including *Magnolia stellata*, *M. wufengensis*, *M. officinalis*, *Sinomanglietia glauca,* and *M. sinostellata* [2,3,4,5,6], tend to grow in coniferous woods and have tiny populations in evergreen broad-leaved forests because of light deficiency (LD) stress. Hence, the shading from taller trees has been generally recognized as an important abiotic stress factor that significantly affects the growth and development of deciduous Magnoliaceae plants [7].

Light is a critical environmental factor that drives photosynthesis [8]. The ability of plants from various ecological niches to capture light effectively is a key factor that determines their survival adaptation in nature [9]. Multiple layers of vegetation can interact in complex ways, with the amount of light that reaches the understory vegetation depending on a variety of factors such as the height and size of the canopy, the species composition of the forest, and the density and arrangement of the trees. Trees with broad leaves generally cast more shade than those with smaller leaves, and taller trees can cast longer shadows. The light intensity perceived by the understory vegetation is influenced by the canopy density of the overstory vegetation. This can have a significant impact on the growth and development of heliophilic plants, which are plants that require a lot of light to grow. Shading caused by a dense canopy cover can be an important abiotic stress factor for these plants, and it can affect their ability to photosynthesize and grow [10,11].

Plants have evolved mechanisms to sense changes in light intensity and quality, including shading caused by the surrounding vegetation. Under a shaded environment, the hypocotyl and petiole of Arabidopsis tend to over-elongate, and the leaf blade area is often reduced [12]. The percentage of the *Vitis vinifera* flowers that fell prematurely was found to increase under shading conditions [13]. Likewise, in *Paeonia lactiflora*, shading caused delays in the initial flowering date, a reduction in flower fresh weight, and a fade in the flower’s color [14]. The chlorophyll-a and -b levels in tea leaves were significantly elevated under shade conditions, allowing them to capture more light energy for photosynthesis [15]. A decline in chlorophyll concentration, which stunts plant growth, was observed as shade intensified over time [16]. Further studies are needed to better understand the connection between light intensity and chlorophyll content.

Chlorophyll is a crucial molecule for photosynthesis in plants. It is a tetrapyrrole compound that contains magnesium and has a porphyrin ring and a long aliphatic side chain (phytol), which plays an important role in the light absorption and photosynthesis of plants [17]. The regulatory networks that control chlorophyll biosynthesis and degradation are complex and involve many genes and signaling pathways [18,19]. The functions of chlorophyll synthase and the regulatory networks that govern chlorophyll biosynthesis have been extensively studied [20,21]. Mutations in genes involved in the chlorophyll biosynthesis pathway can affect the biosynthesis of chlorophyll and result in the altered pigmentation of plant tissues. For example, transgenic Arabidopsis plants that expressed antisense *HEMA1* mRNA showed a conspicuous deficiency in chlorophyll, resulting in yellow leaves [22]. Mutations in other genes involved in the biosynthesis of chlorophyll also result in reduced chlorophyll accumulation. For instance, mutations in genes such as *ChlD* and *ChlI*, which encode subunits of the Mg-chelatase enzyme that is required for chlorophyll biosynthesis, resulted in decreased chlorophyll accumulation and altered leaf pigmentation [23].

Despite the efforts made so far to elucidate the chlorophyll synthase regulatory hierarchy, the mechanisms underlying how they mediate chlorophyll degradation are still largely shrouded in mystery. Leaf yellowing is a prominent feature of leaf senescence, which is a natural process that occurs in plants as they age [24]. Chlorophyll degradation is a critical component of this process as it allows the plant to recycle valuable nutrients and prevent oxidative damage from accumulated chlorophyll in aging tissues [25,26]. The PAO (phophorbide a oxygenase) pathway is a multi-layered regulatory network that controls chlorophyll degradation in plants [27]. The *STAY-GREEN* (*SGR*) gene encodes a Mg-dechelatase, which is a key component of this pathway. The SGR protein interacts with chlorophyll catabolic enzymes (CCEs) and light-harvesting complex II (LHCII) to form the SGR-CCEs-LHCII complex, which catalyzes the conversion of chlorophyll-a to pheophytin-a [28].

The expression of the *SGR* gene is regulated by various plant hormones and abiotic stress signals. For instance, ethylene, abscisic acid, and jasmonic acid are known to play important roles in regulating plant senescence, and their signaling pathways can modulate *SGR* expression. Several transcription factors have also been identified that can bind to the promoter of the *SGR* gene and regulate its expression. For example, EIN3, ABI3, and MYC2/3/4 are transcription factors involved in ethylene, abscisic acid, and jasmonic acid signaling, respectively, and can regulate *SGR* expression in Arabidopsis [29,30,31]. During dark-induced senescence, phytochrome-interacting factors (PIFs) such as PIF4/5 can promote the expression of *EIN3*, *ORE1,* and *ABI5*, which in turn can promote *SGR* expression [32]. Additionally, PIF5 can bind to G-box motifs in the promoter regions of *SGR*, *NYC1*, and *ORE1* and enhance their expression in Arabidopsis [33].

*M. sinostellata* is an endangered species and is listed in the “International Union for Conservation of Nature (IUCN) red list of threatened species” [34]. Previous studies have shown that *M. sinostellata* is particularly sensitive to shading stress, which can compromise its growth and development by affecting plant biological activities such as chlorophyll accumulation, photosynthesis efficiency, and hormone signaling [7,35]. There is a clear knowledge gap about the molecular mechanism of chlorophyll degradation in *M. sinostellata* under LD stress conditions, necessitating further investigation. In this study, we found the chlorophyll degradation gene *MsSGR,* which was significantly up-regulated under LD conditions and served a chlorophyll degradation function. Analysis of the *MsSGR* promoter elucidated that it contains hormone- and light-responsive cis-acting elements and that it responded to LD stress. Yeast two-hybrid (Y2H) screening identified numerous proteins that directly interact with MsSGR, providing vital clues about the molecular pathways involved in LD adaptation in *M. sinostellata*.

## 2. Results

### 2.1. The Light Intensity Received by M. sinostellata Varys Greatly among Different Forest Communities

**Figure 1:**
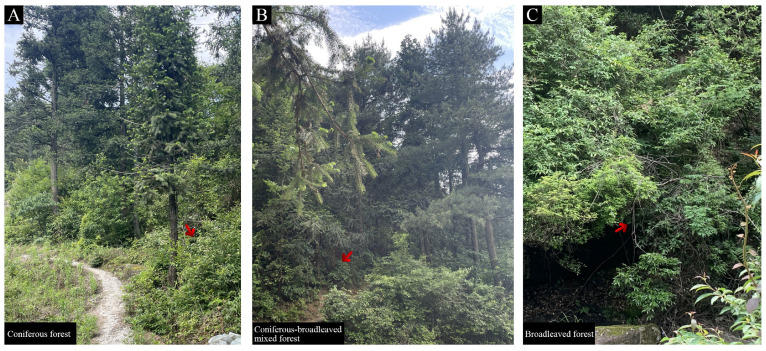
Three typical growth habitats of *M. sinostellata:* (**A**) coniferous forest; (**B**) coniferous-broadleaf mixed forest; and (**C**) broadleaf forest. The red arrow represents the seedlings of *M. sinostellata* in the forest community.

The overstory trees cast shadows onto the understory, reducing the amount of sunlight reaching the deciduous plant there. *M. sinostellata* plants in three distinct habitats, including coniferous forest, broadleaf forest, and mixed forest communities, were analyzed for their relative sensitivity to LD stress. There were substantial variations in light intensity among the three different habitats (Figure 1). In the coniferous forest, the light intensity was about 51,000–57,000 lx; however, in the broadleaf forest and mixed forest communities, the light intensities were in the ranges of 180–250 lx and 400–750 lx, respectively (Table 1). Meanwhile, *M. sinostellata* exhibited diverse phenotypes across different populations. In the broadleaf forest and mixed forest communities, the seedlings of *M. sinostellata* displayed larger leaves and longer stems compared to those in coniferous forest communities (Figure S1). Neither fruits were discernible in the broadleaf forest and mixed forest communities. In the mixed forest community, *M. sinostellata* branches were prostrate with adventive roots (Figure S1B). These observed variations in *M. sinostellata’s* morphology suggest that LD stress caused by the shade of overstory trees exerts a far-reaching impact on the plant’s growth and development.

### 2.2. Light Deficiency Accelerated Leaves Senescence in M. sinostellata

**Figure 2:**
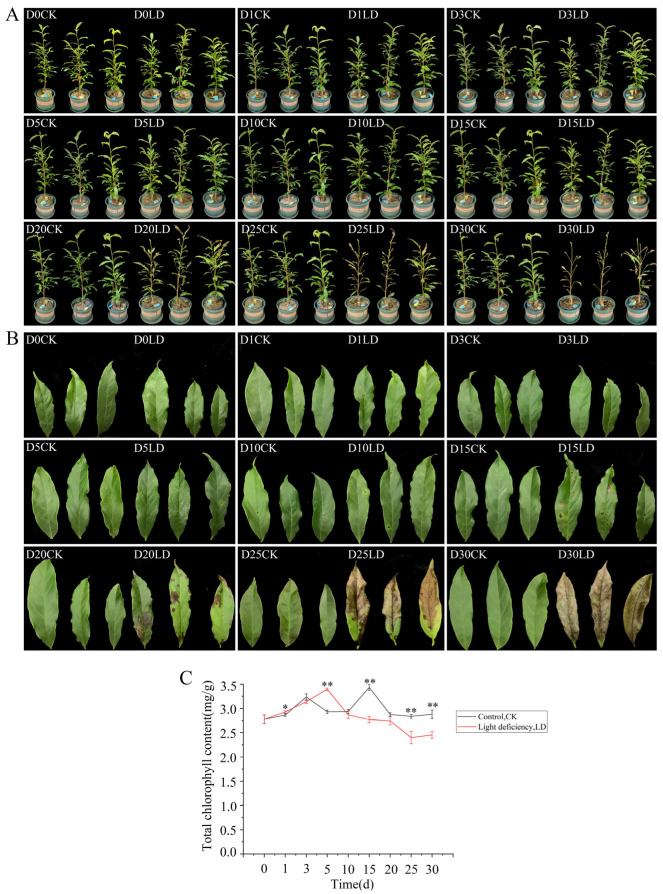
Phenotypic and chlorophyll content changes of *M. sinostellata* under normal and light deficiency (LD) conditions. (**A**) plant shape changes (Control, CK; Light deficiency, LD); (**B**) leaf shape changes; (**C**) chlorophyll content changes of leaves; * represents a significant difference (*p* < 0.05); ** represents a significant difference (*p* < 0.01).

To further clarify the specific influence caused by LD stress in *M. sinostellata*, a field environment simulation experiment was constructed. Under LD conditions, the leaf morphology of *M. sinostellata* altered significantly compared to that under normal light conditions (CK). *M. sinostellata* leaves began to fall after 15 days (d) of light deprivation on the seedlings, accompanied by the appearance of many dark spots. Following 20 d of light deprivation, the dark spots on the leaves of *M. sinostellata* became more noticeable. After 25 d, two-thirds of the leaf surfaces were brown and wilted. After 30 d, practically all of the leaves turned brown and dropped off (Figure 2A,B). Total chlorophyll contents in both LD and control conditions showed an ascending trend followed by a descending trend, but the chlorophyll content was significantly lower in the LD leaves than those in the control from 10 d on (Figure 2C).

### 2.3. Expression Patterns of Genes Involved in Chlorophyll Biosynthesis and Degradation Pathway in M. sinostellata under Light Deficiency Conditions

**Figure 3:**
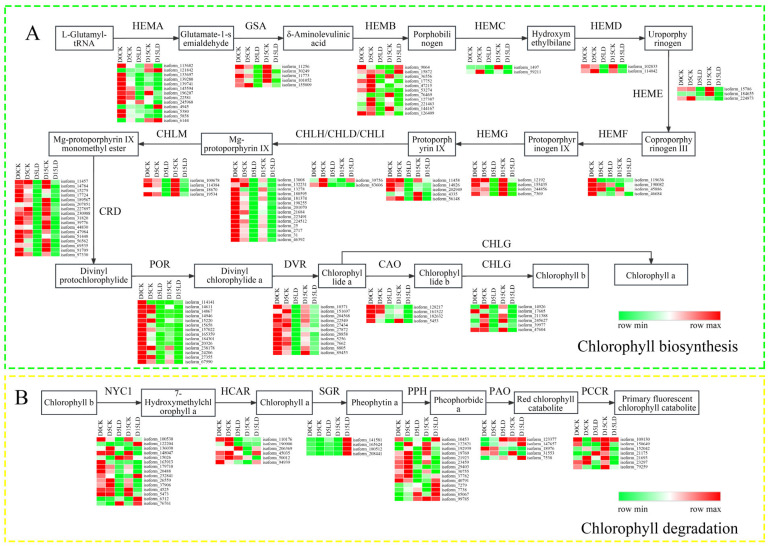
Expression patterns of the genes involved in chlorophyll biosynthesis and degradation pathways in *M. sinostellata* under light deficiency (LD). (**A**) Chlorophyll biosynthesis pathway; (**B**) chlorophyll degradation pathway.

**Figure 4:**
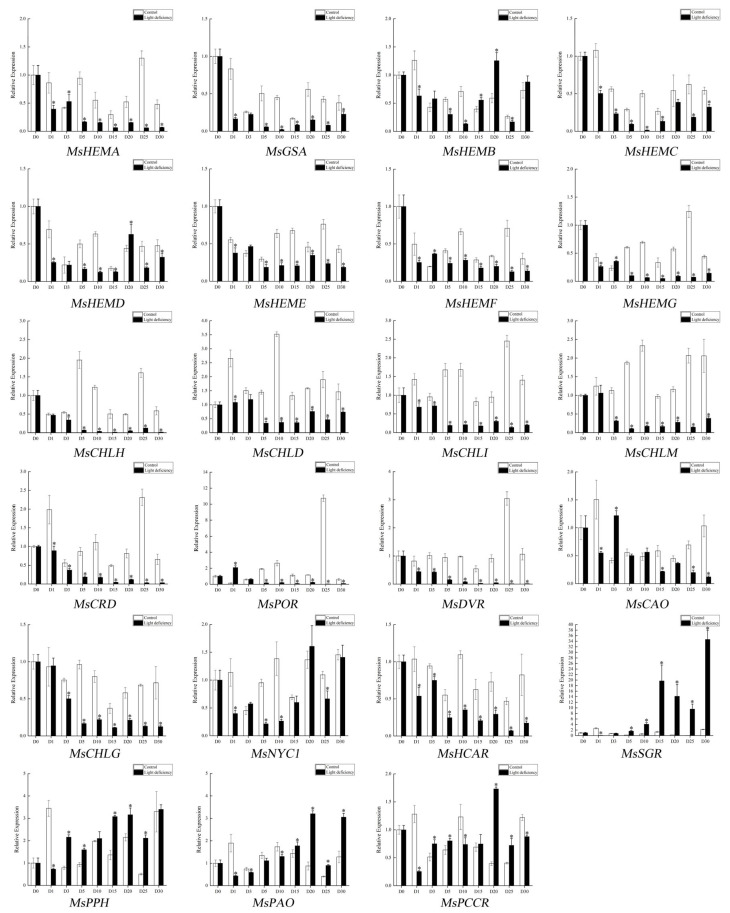
Quantitative reverse transcriptase PCR (qRT-PCR) analysis of chlorophyll biosynthesis and degradation gene under LD conditions; * represents a significant difference (*p* < 0.05).

The expression patterns of genes in chlorophyll biosynthesis and degradation pathways were analyzed to elucidate the molecular processes that underpin chlorophyll depletion under LD conditions. The sequences of genes in chlorophyll biosynthesis and degradation pathways of *M. sinostellata* were similar to the homologous gene sequences in Arabidopsis and rice, and they all contained the same conserved domain, which indicated the same function (Figure S2). As shown in Figure 3A,B, the majority of genes involved in chlorophyll biosynthesis were down-regulated in response to LD treatment, but the majority of genes, with the exception of the *MsSGR* gene, did not exhibit significant expression changes. The expression pattern of genes was verified by the quantitative reverse transcriptase polymerase chain reaction (qRT-PCR) analysis (Figure 4), which revealed that the majority of genes involved in chlorophyll biosynthesis, except for *MsHEMB,* were down-regulated under LD conditions. Meanwhile, *MsSGR*, *MsPPH*, and *MsPAO* were up-regulated, and the up-regulated trend of the *MsSGR* gene was the most significant under LD conditions, indicating that *MsSGR* plays a vital role in the shade tolerance response of *M. sinostellata* under LD conditions.

### 2.4. Identification and Characterization of MsSGR

**Figure 5:**
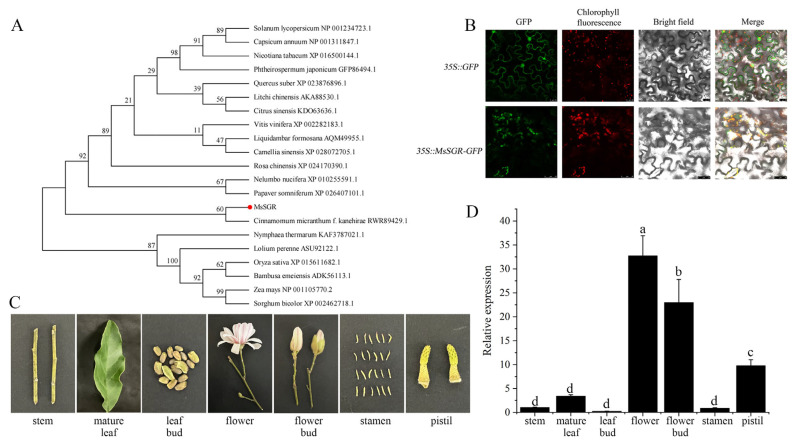
Identification and characterization of *MsSGR*. (**A**) Phylogenetic tree analysis of SGR protein. The amino acid sequence used in the analysis was listed as follows: *Litchi chinensis* (AKA88530.1), *Vitis vinifera* (XP_002282183.1), *Nelumbo nucifera* (XP_010255591.1), *Liquidambar formosana* (AQM49955.1), *Rosa chinensis* (XP_024170390.1), *Nicotiana tabacum* (XP_016500144.1), *Citrus sinensis* (KDO63636.1), *Cinnamomum micranthum* (RWR89429.1), *Quercus suber* (XP_023876896.1), *Phtheirospermum japonicum* (GFP86494.1), *Camellia sinensis* (XP_028072705.1), *Solanum lycopersicum* (NP_001234723.1), *Capsicum annuum* (NP_001311847.1), *Oryza sativa* (XP_015611682.1), *Lolium perenne* (ASU92122.1), *Nymphaea thermarum* (KAF3787021.1), *Papaver somniferum* (XP_026407101.1), *Zea mays* (NP_001105770.2), *Sorghum bicolor* (XP_002462718.1), and *Bambusa emeiensis* (ADK56113.1). (**B**) Subcellular localization of MsSGR in tobacco leaves; (**C**) the tissues of *M.sinostellata* seedlings; (**D**) tissue-specific expression of *MsSGR* gene, different letters represent significant differences (*p* < 0.05).

*MsSGR* gene was amplified by PCR using *M. sinostellata* leaf cDNA as a template. Multiple protein alignments of the MsSGR and its orthologs in various other plant species showed that MsSGR contains the hallmark STAY-GREEN domain and the cysteine-rich motif, indicating that MsSGR is a member of the SGR subfamily but not the SGRL subfamily (Figure S3). The phylogenetic tree revealed that SGR homologs clearly fall into two categories: monocotyledonous plants and dicotyledonous plants, indicating that SGR conforms to the laws of evolution (Figure 5A). In addition, *MsSGR* gene was expressed in the stem, leaf, leaf bud, flower, flower bud, stamen, and pistil, as determined by tissue-specific expression. Expression was the most abundant in the flower and flower bud, followed by pistil, leaf, stem, stamen, and leaf bud (Figure 5C,D). To determine if chloroplasts are the functional location of MsSGR, *Agrobacterium tumefaciens* cells harboring the 35S::GFP or 35S::MsSGR-GFP constructs were infiltrated into tobacco leaves. As illustrated in Figure 5B, in the leaf discs infiltrated with 35S::GFP, the GFP fluorescence signal was present in the nucleus and the cytomembrane but not the chloroplasts. In contrast, GFP fluorescence was only visible in the chloroplasts in the leaf discs infiltrated with 35S::MsSGR-GFP (Figure 5B). Therefore, MsSGR was conceivably located in the chloroplasts.

### 2.5. MsSGR Induced Chlorophyll Degradation in Arabidopsis and Tobacco

**Figure 6:**
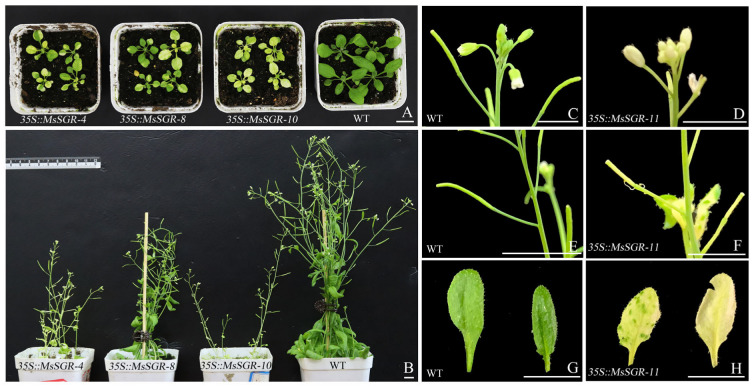
*MsSGR* induces chlorophyll degradation in Arabidopsis; (**A**) represents the plant shapes of Arabidopsis at 15 days (d); (**B**) represents the plant shapes of Arabidopsis at 35 d; (**C**,**E**,**G**) represent inflorescences, pods and leaves of wild-type (WT) Arabidopsis; and (**D**,**F**,**H**) represent inflorescences, pods, and leaves of *MsSGR* overexpressed Arabidopsis, respectively, bar = 1 cm.

To investigate how *MsSGR* functions in the chlorophyll degradation process, the *MsSGR* gene was cloned and integrated into the plant expression vector pORE-R4 (Figure S4). *MsSGR* was overexpressed in transgenic Arabidopsis and transiently expressed in tobacco leaves. The transgenic Arabidopsis plants displayed conspicuous alterations in morphology compared to the wild-type (WT) lines. Notably, overexpression of the *MsSGR* gene in Arabidopsis resulted in pre-withered rosette leaves (Figure 6A,G,H). In addition, the transient expression of the *MsSGR* gene in tobacco leaves caused severely withered maculas in leaves and induced the chlorophyll degradation process (Figure S5). In addition to significant phenotypic aberrations in the leaves, transgenic Arabidopsis exhibited significantly stunted development, reaching a maximum height of 12–13 cm as opposed to 30 cm in WT (Figure 6B), and forming hypogenetic inflorescences with yellow sepals and peduncles (Figure 6C,D). The structures of hypogenetic inflorescences further developed into short and abortive siliques that bear few viable seeds (Figure 6E,F). Taken together, these findings demonstrate that *MsSGR* plays a crucial role in the chlorophyll degradation process and has a profound effect on plant growth and development.

### 2.6. Light Deficiency Promotes MsSGR Promoter Activity

**Figure 7:**
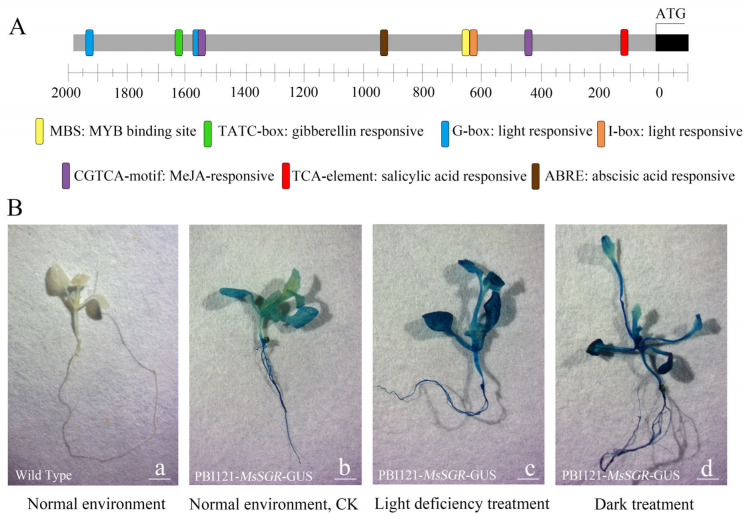
Analysis of *MsSGR* promoter; (**A**) cis-acting element analysis of the *MsSGR* promoter; (**B**) GUS enzyme activity analysis under light deficiency. (a) represents wild type in normal environment; (b–d) represent *MsSGR::GUS* transgenic Arabidopsis in normal environment (CK), light deficiency treatment and dark treatment, respectively, bar = 2 mm.

The putative *MsSGR* promoter, spanning 1944 bp in length, was cloned using PCR and verified using sequence analysis (Figure S6A). GUS enzyme activity analysis revealed the regulatory activity of the *MsSGR* promoter (Figure S6B,C). The analysis of the cis-acting elements by searching the PlantCARE database revealed that the *MsSGR* promoter encompasses four hormone-responsive elements, including gibberellin, abscisic acid, salicylic acid, and MeJA, as well as two light-responsive elements, including the I-box and the G-box. It is tempting to infer that *MsSGR* gene expression is regulated by light signals and various plant hormones (Figure 7A). To further investigate the regulatory mechanisms of the *MsSGR* gene in response to LD stress, the putative *MsSGR* promoter sequence was used to drive β-glucuronidase (GUS) expression by generating a MsSGR::GUS construct that was used to transform Arabidopsis. It appeared that the GUS staining was discernible in the leaf, hypocotyl, and root in the transgenic lines (Figure 7B(a,b)). In response to LD and dark treatments, the hypocotyls and petioles of the transgenic lines were overtly elongated and displayed a significantly higher level of GUS staining in leaves, hypocotyls, and petioles compared to the CK, indicating the regulatory response of the *MsSGR* promoter to LD treatment (Figure 7B(b–d)).

### 2.7. Potential Interacting Proteins of MsSGR by Y2H Analysis

**Figure 8:**
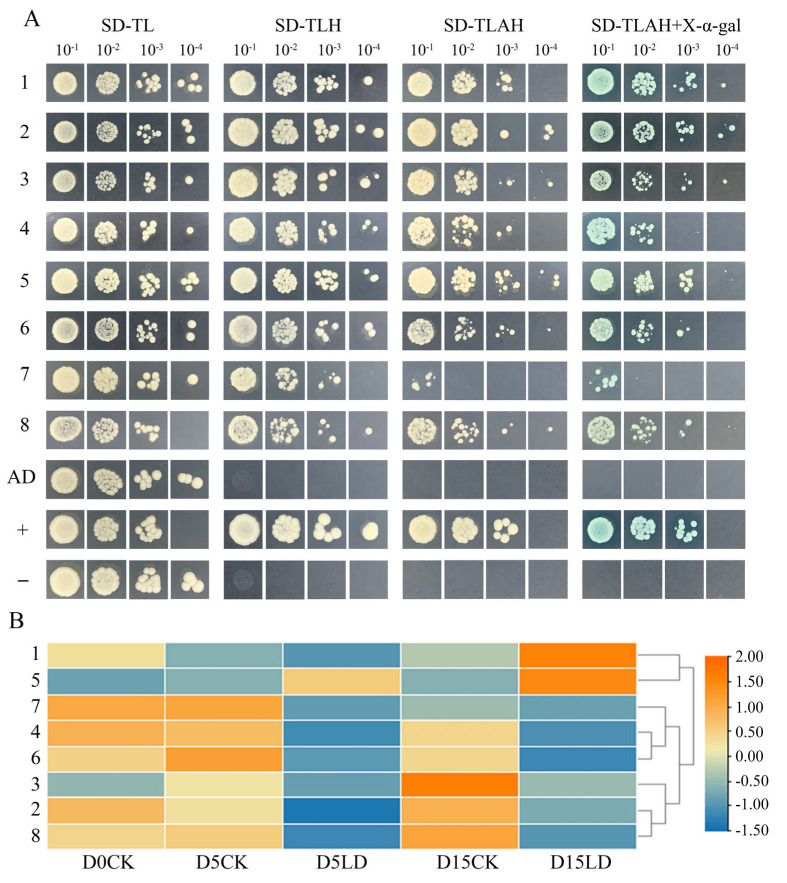
Yeast two-hybrid (Y2H) screening of the putative chloroplastic proteins that are interacting with MsSGR. (**A**) Screened colonies grew on medium SD-Trp-Leu, SD-Trp-Leu-His, SD-Trp-Leu-His-Ade, and SD-Trp-Leu-His-Ade+x-α-gal. “AD” represents the self-activation group (pGBKT7-MsSGR+pGADT7); “+” represents the positive control (pGBKT7-T+pGADT7-P53); and “−” represents the negative control (pGBKT7-T+pGADT7-Lam). Yeast liquid dilution concentrations: 10^−1^, 10^−2^, 10^−3^, and 10^−4^. (**B**) The heatmap of coding chloroplastic genes expression patterns under light deficiency treatment.

In order to clarify the potential molecular mechanisms of MsSGR, a Y2H library with a capacity of 5.56 × 10^7^ CFU was constructed (Figure S7). MsSGR exhibited no self-activation or cell toxicity in yeast cells (Figure S8). The screening among the Y2H library produced 74 colonies, which were growing strongly on SD-Trp-Leu-His. These *HIS*^+^ positive colonies were sequenced and contained sequences encoding 41 proteins. In order to further determine the positive interaction between 41 proteins and MsSGR, these colonies were transferred to the SD-Trp-Leu-Hi-Ade and SD-Trp-Leu-His-Ade+x-α-gal to detect whether the *Ade* and *MEL1* reporter genes were activated. A total of 24 proteins directly interacted with MsSGR, eight of which were chloroplast proteins (Table 2, Figure 8A). To elucidate the potential shading response mechanisms, the expression patterns of eight interacting chloroplast proteins under light deficiency conditions were examined. The results showed that the expression levels of eight chloroplast genes significantly responded to LD stress, of which six genes were significantly downregulated and one was highly upregulated. In particular, a gene encoding polyphenol oxidase was downregulated at 5 d and accumulated highly at 15 d (Figure 8B).

## 3. Discussion

LD is one of the major abiotic factors affecting plant growth and development as it is a crucial source of energy for photosynthesis [36,37]. Without sufficient light, plants may not be able to synthesize enough food to support their growth and development, leading to various physiological and morphological changes, as observed in *M. sinostellata* in the present study. It was emphatically shown that the light intensity of *M. sinostellata* in the mixed forest and broadleaf forest was far below that in the coniferous forest (Table 1). Compared with seedlings grown in the coniferous forest, the *M. sinostellata* seedlings grown in the mixed forest and broadleaf forest had larger leaves and longer stems (Figure S1), which is consistent with the plant shade avoidance syndrome (SAS), which is a set of physiological and morphological changes observed in plants in response to reduced light availability or shading by neighboring plants [38]. When plants are exposed to shade, they perceive changes in the light spectrum, particularly a decrease in the ratio of red to far-red light. This triggers a series of responses, including elongation of stems and petioles, increased leaf area, reduced branching, and altered flowering time, all of which are thought to enhance the plant’s ability to capture more light. *M. sinostellata* did not produce any fruits in the broadleaf forest and mixed forest communities. In the mixed forest community, the branches of *M. sinostellata* were observed to be prostrate and have adventive roots (Figure S1B). Furthermore, in conditions of light deprivation, the leaves of *M. sinostellata* rapidly wilt and senesce, with almost all of the leaves falling off under an LD environment, indicating that *M. sinostellata* is particularly sensitive to LD (Figure 2A,B). Such observations are congruent with previous reports in many other plant species under LD conditions, such as hypocotyl elongation in Arabidopsis and tomato [12,39], increases in plant height, canopy perimeter, and canopy volume in lemon trees [40], reduction in the number of flower buds, and a delay in floral transition in Lisianthus [41]. Based on the evidence presented, it is conceivable that LD has a significant impact on the growth, development, and reproduction of *M. sinostellata*, which may have played a major role in the endangerment of this species in its natural habitat.

It is well-recognized that leaf yellowing is a classic symptom of leaves senescence, which is primarily caused by chlorophyll degradation [42]. Chlorophyll degradation is a natural process that occurs in plants during various stages of their life cycle, such as senescence, ripening, and stress responses. To prevent free chlorophyll from generating additional photo-oxidative damage, plants must rapidly break down these molecules as a survival strategy [43]. Numerous plants, including rice and Arabidopsis, exhibit a senescence phenotype characterized by leaf yellowing under darkness to promote chlorophyll degradation [44,45]. In this study, the total chlorophyll content showed a general increasing trend followed by a descending trend, although the descending trend of the total chlorophyll content was not obvious compared with the leaf color changes. Because the weight changes caused by water loss in senescent leaves will affect the measurement of chlorophyll content based on weight (mg g^−1^), it is better to use leaf area-based measurement (mg cm^−2^) to measure the chlorophyll content of senescent leaves [46]. However, the descending trend of total chlorophyll content was still significant (*p* < 0.01) based on weight measurement (Figure 2C). The decline in the content of chlorophyll content impaired *M. sinostellata*’s ability to capture light energy. In addition, LD induced the downregulation of almost all the genes involved in chlorophyll biosynthesis, whereas the *MsSGR* gene was significantly upregulated (Figure 3 and Figure 4), indicating that *M. sinostellata* reduces leaf chlorophyll content by inhibiting chlorophyll biosynthesis and accelerating chlorophyll degradation to prevent chlorophyll from causing more photooxidative damage to cells in LD. Overall, *M. sinostellata* responded to LD by reducing the chlorophyll content in its leaves. However, there was a possibility that the decrease in chlorophyll content impaired photosynthesis in *M. sinostellata.*

As a key gene in the chlorophyll degradation pathway, the *SGR* gene encodes a Mg-dechelatase, which catalyzes the removal of the central magnesium ion from chlorophyll, leading to its breakdown and the subsequent degradation of the chloroplast [47]. In this study, *MsSGR* isolated from *M. sinostellata* was determined to be a chloroplast-specific protein through subcellular localization (Figure 5B), which is consistent with its orthologs in Arabidopsis and *Camellia sinensis* [28,48]. Furthermore, protein structure analysis revealed that MsSGR does not have any transmembrane structure (Figure S9), suggesting that it is most likely localized in the chloroplast stroma, which is the fluid-filled space inside the chloroplasts where many of the biochemical reactions associated with photosynthesis and chlorophyll degradation occur. Overexpression of *MsSGR* caused leaf yellowing in the transgenic Arabidopsis and a decrease in the chlorophyll content of tobacco that was transiently expressing *MsSGR* (Figure 6A,G,H and Figure S5). These results were consistent with previous studies, which demonstrated the functional role of the *SGR* gene in chlorophyll degradation [48]. In addition, yellow stems, yellow inflorescences, and abortive fruit pods were also observed in transgenic Arabidopsis overexpressing *MsSGR* (Figure 6B–F), lending further credence to the assumed role of the *MsSGR* gene in accelerating chlorophyll degradation. Furthermore, the spatial differentiation of *MsSGR* in *M. sinostellata* tissues suggests its functional diversity and specificity, which may have evolved through gene duplication. Notably, *MsSGR* was observed in higher expression in flower and flower bud, which is consistent with previous reports in Arabidopsis [49]. The chlorophyll degradation gene *PAO* was also expressed highly in flowers [25]. In *Magnolia* plants, cyanidin and peonidin make the flower petals appear red-purple and purple, respectively, and the flavonols perform as auxiliary pigments [50]. It is reasonable to speculate that the high expression of *MsSGR* might promote chlorophyll degradation in flowers. Nonetheless, further research is needed.

Analysis of the *MsSGR* promoter sequence revealed the presence of four hormone-responsive elements and two light-responsive cis-acting elements (Figure 7A). The results indicated that the *MsSGR* gene is regulated by a complex regulatory network. As indicated by previous works, *SGR* is controlled by plant senescence hormone and transcription factor families [29,31,51]. Meanwhile, *SGR* also responds to changes in light signals, phytochrome interacting factors PIF4 can bind to the promoter of *SGR* to promote its expression in Arabidopsis [45]. In this study, a greater deposition of GUS stain was observed in the hypocotyl, leaf, and petiole of *MsSGR::GUS* transgenic lines under LD conditions, indicating that the promoter of *MsSGR* is responsive to LD stress (Figure 7B). Taken together, *MsSGR* was found to be an important gene in the chlorophyll degradation process of *M. sinostellata* in response to LD stress.

Previous studies have focused mostly on how SGR regulates chlorophyll degradation [52,53,54]. SGR is able to bind the light-harvesting complex II (LHCII) protein and recruit chlorophyll catabolic enzymes (CCEs) to form the SGR-CCEs-LHCII complex [28]. In *Camellia sinensis*, CsSGR can generate a CsSGR-CssHSP-CsLHCII protein complex to regulate albinism [48]. In addition, tomato SlSGR1 directly interacts with the carotenoid biosynthesis enzyme SlPSY1 and inhibits its activity in order to regulate tomato lycopene accumulation [55]. Therefore, not only is SGR engaged in chlorophyll degradation but it also contributes to the carotenoid biosynthesis pathway, indicating that SGR has a diverse range of functions. In the present study, a total of eight potential chloroplast proteins that interacted with MsSGR were identified (Table 2, Figure 8A). Among these proteins, polyphenol oxidase is the key enzyme in the enzymatic browning of fruits and vegetables [56]; uroporphyrinogen decarboxylase encoded by the *HEME* gene is involved in the chlorophyll biosynthesis pathway [57]. The transcriptome analysis revealed that genes encoding eight chloroplast proteins exhibited differential expression in response to LD stress (Figure 8B). It is envisaged that the eight chloroplast proteins are likely to be involved in regulating chlorophyll degradation by interacting with MsSGR in *M. sinostellata* in response to LD stress. Therefore, the findings of Y2H screening in this study can provide valuable information about protein–protein interactions and potential molecular mechanisms involved in the chlorophyll degradation process in response to LD stress. Further research could then be directed towards investigating these interactions and mechanisms to gain a better understanding of MsSGR’s role in an intricate molecular network involving many other chloroplast-localized proteins under LD stress.

## 4. Material and Methods

### 4.1. Light Intensity Measurement in Habitats of M. sinostellata

Light intensity was measured in the coniferous forest, broadleaf forest, and mixed forest communities where *M. sinostellata* grew. In each community, three *M. sinostellata* seedlings were chosen as measurement stations. The coordinates for each measurement station were recorded by global positioning system (GPS) (Garmin, Olathe, KS, USA). The light intensity was measured in luminous flux using a Digital Luxmeter ZDS-10 (Shanghai Jiading Xuelian Instrument, Shanghai, China), with three replicates for each measurement.

### 4.2. Plants Materials and Light Deficiency Treatments

The 3-year-old grafted *M. sinostellata* seedlings were collected from the Shengzhou Magnolia base, Shaoxing, Zhejiang Province, China. In this experiment, these seedlings were evenly placed in a climate chamber at 25 °C, 50% humidity, 14/10 h light/dark photoperiod, and 648 µmol m^−2^·s^−1^ PAR (photosynthesis active radiation). To simulate the LD stress conditions caused by shading in natural environments, one part of *M. sinostellata* seedlings was placed under an LD stress treatment condition (16.2 µmol m^−2^·s^−1^ PAR, LD) that was set up using a black shade net and several bamboo poles, and the other part of the seedlings was placed in a normal condition (648 µmol m^−2^·s^−1^ PAR, CK) in a climate chamber. The light intensity was measured in luminous flux with a Digital Luxmeter ZDS-10 (Shanghai Jiading Xuelian Instrument, Shanghai, China) and converted the light intensity from LUX to PAR as previously described [58]. All other experimental conditions were maintained the same for both LD stress treatment and CK, with three biological replicates. Leaf samples were collected from the seedlings in LD treatment and CK groups at 0, 1, 3, 5, 10, 15, 20, 25, and 30 d following the treatment. The samples of leaf, leaf bud, flower, flower bud, stamen, and pistil were collected from the *M. sinostellata* seedlings. Each sample was collected from three seedlings and each collection was repeated three times as biological replicates. All samples were stored at −80 °C for further experimentation.

### 4.3. Determination of Chlorophyll Content

The chlorophyll was extracted from 100 mg leaves using 10 mL of 95% ethanol. The extracts were filtered and analyzed with a Shimadzu UV2700 spectrophotometer (Shimadzu, Kyoto, Japan), and the absorbances were recorded at both 649 and 665 nm. Total chlorophyll content (mg g^−1^) was estimated using the method previously described by Welburn and Lichtenthaler [59]. All the experiments were performed in triplicate.

### 4.4. Transcriptome Analysis of Chlorophyll Metabolism Pathway Genes

The transcriptome data are derived from our previously published study [7]. The genes involved in the chlorophyll biosynthesis and degradation pathways were mainly retrieved through the Kyoto Encyclopedia of Genes and Genomes (KEGG) (map00860, Porphyrin Metabolism) pathway enrichment analysis. Heatmaps of gene expression were produced using Morpheus (Morpheus, https://software.broadinstitute.org/morpheus, accessed on 27 May 2021).

### 4.5. RNA Extraction, cDNA Synthesis, and DNA Preparation

Total RNA was extracted from an RNAprep Pure Plant Plus Kit (Tiangen, Beijing, China) following the manufacturer’s instructions. The integrity of RNAs was confirmed using 1% agarose gel electrophoresis, and the purity and concentration of the total RNAs were analyzed using a NanoDrop spectrophotometer (Thermo Fisher Scientific, Waltham, MA, USA). The total RNAs were converted to cDNAs using the PrimeScriptTM^RT^ Master Mix (Takara, Tokyo, Japan) following the manufacturer’s instructions. Total DNA was extracted from a FastPure Plant DNA Isolation Mini Kit (Vazyme, Nanjing, China) following the manufacturer’s instructions.

### 4.6. Expression Analysis of Chlorophyll Biosynthesis and Degradation Gene in Light Deficiency Stress and MsSGR in Different Tissues

The expression patterns of chlorophyll biosynthesis and degradation genes and *MsSGR* in different tissues were assayed using qRT-PCR and the primers were designed using Primer 5.0 (Table S1). The common isoform sequences were used to design primers for qRT-PCR (Figure S10). The qRT-PCR experiment was conducted using BCG qPCR Master Mix (Beijing Baikaiji Biotechnology, Beijing, China) on a LightCycler^®^ 480 II (Roche Applied Science, Penzberg, Germany). The qRT-PCR program was as follows: 95 °C for 2 min, 40 cycles of 95 °C for 15 s, and 60 °C for 40 s. *EF1*-*α* was selected as the reference gene to normalize the gene expression, and the relative gene expression was determined using the 2^−ΔΔCt^ method [60]. All the qRT-PCR analysis experiments were performed in triplicate. The bar charts of gene expression were generated using Origin 2018 (OriginLab Corporation, Northampton, MA, USA), and SPSS 24.0 (SPSS Inc., Chicago, IL, USA) was used to analyze statistical significance.

### 4.7. Cloning of the MsSGR Gene and Bioinformatics Analysis

The *MsSGR* full-length sequence was isolated from our previous transcript data, and primers were designed on both sides of the *MsSGR* open reading frame (ORF). PCR amplification was performed using the primers listed in Table S1. The PCR program was as follows: 95 °C for 3 min, 35 cycles of 95 °C for 15 s, 58 °C for 15 s, and 72 °C for 1 min; with a final extension at 72 °C for 5 min. The purified PCR product was ligated into the pMD-18T vector (Takara, Tokyo, Japan) and verified using DNA sequencing. Multiple sequence alignments of amino acid sequences were performed using DNAMAN 6.0 (Lynnon Biosoft, San Ramon, CA, USA). Phylogenetic trees were generated using MEGA 6.0 (Koichiro Tamura, Tokyo, Japan) based on the neighbor-joining method (1000 bootstrap replicates). Homologous protein sequences of SGR were all downloaded from NCBI (https://www.ncbi.nlm.nih.gov/, accessed on 18 August 2023). The protein transmembrane regions were predicted using TMHMM 2.0 (https://services.healthtech.dtu.dk/services/TMHMM-2.0/, accessed on 16 January 2023).

### 4.8. MsSGR Subcellular Localization and Overexpression in Arabidopsis and Tobacco

The binary vector pORE_R4 containing GFP was used in this experiment. The open reading frame (ORF) of *MsSGR* without a stop codon was amplified by using the primers listed in Table S1. The PCR products were digested with XhoI and ClaI and ligated into the corresponding sites of the pre-digested pORE_R4 to generate the expression vector 35Spro::MsSGR::GFP by using the ClonExpress^®^ II One Step Cloning kit (Vazyme, Nanjing, China). The vector was transformed into *A. tumefaciens* strain GV3101 Chemically Competent Cell (Shanghai Weidi Biotechnology, Shanghai, China), which was used to infiltrate the tobacco leaves. After incubation for 12 h in the dark followed by 48 h under light, GFP fluorescence was observed with a Leica TCS SP8DLS confocal microscope (Leica, Nussloch, Germany). The excitation wavelength for GFP was 488 nm, and the emission wavelength was 498–538 nm. The excitation wavelength for chloroplasts was 552 nm, and the emission wavelength was 640–720 nm. Arabidopsis was transformed with the inflorescence-dip method and selected on Kanamycin-containing 1/2 MS medium.

### 4.9. MsSGR Promoters Clone and Activity Analysis

To isolate the promoter sequence of *MsSGR*, its orthologous sequence, *MbSGR*, was first retrieved from the *Magnolia biondii* genome database [61]. A set of PCR primers was designed based on the promoter sequence of *MbSGR* (Table S1). The putative promoter sequence of the *MsSGR* gene was amplified by PCR using the genomic DNA derived from *M. sinostellata* leaves as a DNA template and verified by DNA sequencing and sequence alignment with the *MbSGR* promoter. The cis-acting elements were predicted using PlantCARE (http://bioinformatics.psb.ugent.be/webtools/plantcare/html/, accessed on 6 August 2021). The *MsSGR* promoter fragment was directionally cloned into the *Hind III* and *XbaI* sites of the binary vector pBI121-GUS replacing the 35S CaMv promoter. The ClonExpress^®^ II One Step Cloning kit (Vazyme) was used to generate a MsSGR::GUS vector. This vector was introduced into *A. tumefaciens* GV3101 (Shanghai Weidi Biotechnology, Shanghai, China), which was used to infiltrate tobacco leaves. *A. tumefaciens* cells containing the pBI121-GUS vector with 35S CaMv promoter served as a positive control, and the cells without any vector were used as a negative control. GUS histochemical staining was performed using the GUS Stain kit (Coolaber, Beijing, China). Arabidopsis was transformed with the inflorescence-dip method and selected on the Kanamycin-containing 1/2 MS medium. Transgenic Arabidopsis plants were subjected to LD stress of 16.2 µmol m^−2^·s^−1^ PAR or a completely dark environment.

### 4.10. Construction of Yeast Two-Hybrid (Y2H) pGADT7 Library

Total RNA was extracted from the sample of leaves and leaf buds derived from *M. sinostellata* using the Trizol method (Invitrogen, Carlsbad, CA, USA), and mRNA was purified from total RNA using oligo (dT) magnetic beads (Vazyme, Nanjing, China). Subsequently, mRNA was reverse-transcribed into single-strand cDNA, and the cDNA was amplified and purified. TRIMMER-2 cDNA normalization kit (EVROGEN, Moscow, Russia) was used to construct homogenized cDNA and Clontech CHROMA SPIN™+TE-1000 Columns (Takara, Tokyo, Japan) was usedto remove small fragments. The double-stranded cDNA was recombined into pGADT7 and then transformed into TOP10 competent cells (Shanghai Weidi Biotechnology, Shanghai, China). Finally, the capacity of two-hybrid (Y2H) pGADT7 library capacity was identified, and it used HighPure Maxi Plasmid Kit (Tiangen, Beijing, China) to extract library plasmid.

### 4.11. Yeast Two-Hybrid (Y2H) Screen of MsSGR Protein

The ORF of *MsSGR* was inserted into the bait vector pGBKT7 to generate the BD fusion vector, pGBKT7-*MsSGR*, which was co-transformed with pGADT7 (AD) into the yeast strain AH109 (Shanghai Weidi Biotechnology) using heat shock transformation. Positive transformants containing both AD and BD were selected on a synthetic defined (SD) medium (Coolaber, Beijing, China) lacking Trp and Leu (SD-Trp-Leu). Following PCR confirmation, the positive transformants were inoculated on an SD medium lacking Trp, Leu, and His (SD-Trp-Leu-His), an SD medium lacking Trp, Leu, His, and Ade (SD-Trp-Leu-His-Ade), and an SD medium lacking Trp, Leu, His, and Ade but adding x-α-gal (SD-Trp-Leu-His-Ade+x-α-gal), to test whether MsSGR can self-activate the expression of His, Ade, and MEL1 reporter genes. Subsequently, the BD (pGBKT7-MsSGR) and AD (Y2H pGADT7 library) were co-transformed into the yeast strain AH109 (Shanghai Weidi Biotechnology), and the cells were cultured on an SD medium lacking Trp, Leu, and His (SD-Trp-Leu-His). The positive transformants were selected for PCR and sequenced. Finally, the positive transformants were inoculated on SD-Trp-Leu, SD-Trp-Leu-His, SD-Trp-Leu-His-Ade, and SD-Trp-Leu-His-Ade with added x-α-gal to further determine the potentially interacting proteins with MsSGR.

## 5. Conclusions

The present study revealed the effect of LD stress on the chlorophyll metabolism pathway of *M. sinostellata* and the role of *MsSGR* in regulating chlorophyll degradation in *M. sinostellata* under LD stress. We also studied the interactive network of the MsSGR protein and identified eight chloroplast proteins interacting with MsSGR. Overall, these findings highlight the fact that LD stress can have a significant impact on the growth of *M. sinostellata* by affecting the chlorophyll metabolism pathway. This study also contributes to our understanding of the endangerment mechanisms of wild Magnoliaceae species and provides a theoretical basis for developing conservation strategies. By identifying the interactive network of MsSGR, this study can provide insights into potential targets for interventions aimed at mitigating the effects of LD stress on Magnoliaceae species.

## Figures and Tables

**Table 1:** Geographic information and light intensity of three forest communities of *M. sinostellata*.

Type	Side	Latitude	Longitude	Altitude	Aspect	Slope	Light Intensity/(Lx)		
Coniferous forest	A	28°18′58″ N	119°49′12″ E	990	318° Northwest	16°	60,000	56,000	53,000
	B	28°18′58″ N	119°46′12″ E	1000	310° Northwest	11°	52,000	51,000	50,000
	C	28°12′59″ N	119°46′11″ E	980	20° North	22°	48,000	54,000	52,000
Coniferous-broadleaf mixed forest	A	28°12′51″ N	119°46′10″ E	960	340° North	42°	501	360	350
	B	28°12′51″ N	119°46′10″ E	960	358° North	41°	671	806	732
	C	28°12′51″ N	119°46′11″ E	970	344° North	30°	420	321	433
Broadleaf forest	A	28°10′59″ N	119°49′21″ E	990	88° East	25°	207	238	212
	B	28°10′58″ N	119°49′21″ E	980	74° East	23°	273	204	196
	C	28°10′58″ N	119°49′21″ E	990	87° East	40°	150	214	197

**Table 2:** Y2H Screened colonies information.

No.	GenBank No.	Homologous Protein	Clone Numbers
1	EHA8589896.1	polyphenol oxidase, chloroplastic	4
2	XP_030942398.1	peptidyl-prolyl cis-trans isomerase FKBP13, chloroplastic	4
3	XP_020695605.1	ribulose bisphosphate carboxylase small chain, chloroplastic	4
4	XP_034688430.1	PHOTOSYSTEM I ASSEMBLY 2, chloroplastic	1
5	RWR91980.1	multiple organellar RNA editing factor 8, chloroplastic/mitochondrial-like	1
6	RWR94664.1	ATP-dependent Clp protease proteolytic subunit-related protein 3, chloroplastic	1
7	RWR83419.1	protein PTST, chloroplastic	1
8	XP_029123969.1	uroporphyrinogen decarboxylase, chloroplastic	1

## Data Availability

The RNAseq data that support the findings of this study were deposited in the National Center for Biotechnology Information (NCBI) with the accession number PRJNA770262.

## Supplementary Materials

The following supporting information can be downloaded at: https://www.mdpi.com/article/10.3390/ijms24108510/s1.
