# Supplementary material for: STAY-GREEN Accelerates Chlorophyll Degradation in Magnolia sinostellata under the Condition of Light Deficiency
Source: Int J Mol Sci. 2023 May 9;24(10):8510. doi: 10.3390/ijms24108510 (PMC10218395; doi:10.3390/ijms24108510)
Supplement: Supplementary file 1 [file ijms-24-08510-s001.zip › ijms-2266116-supplementary.pdf]

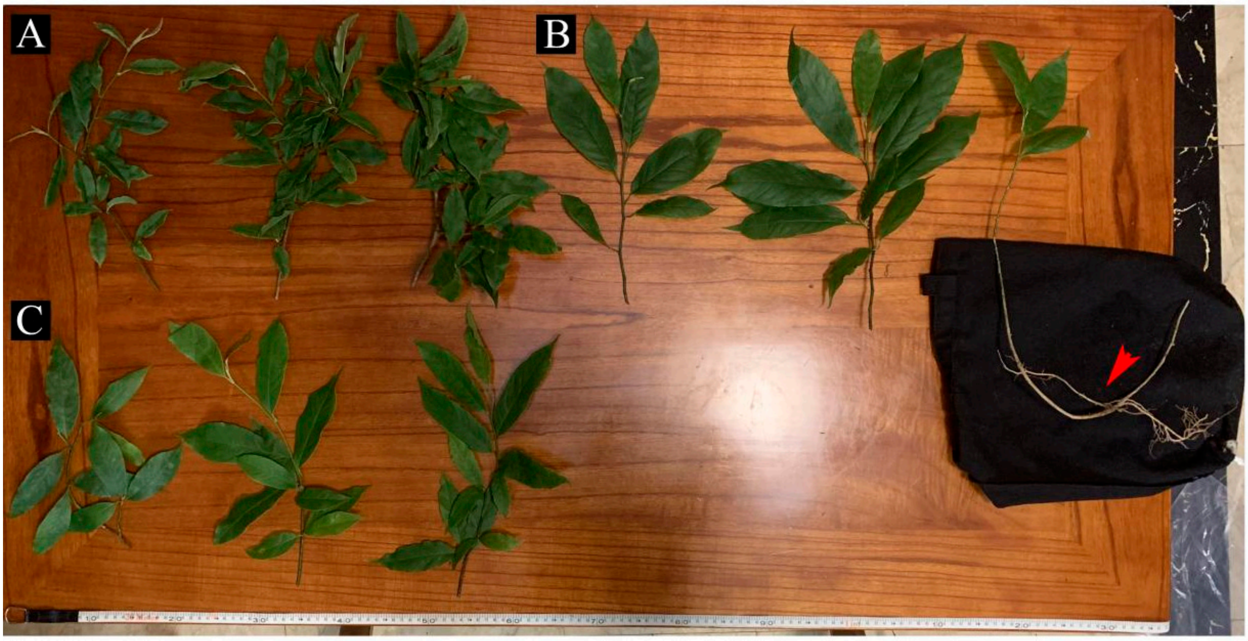

Figure S1. The phenotype of *Magnolia sinostellata* seedlings in wild. **(A)** Coniferous forest. **(B)** Coniferous-broadleaved mixed forest. **(C)** Broadleaved forest. The red arrow represents the adventive roots in branches of *M. sinostellata*

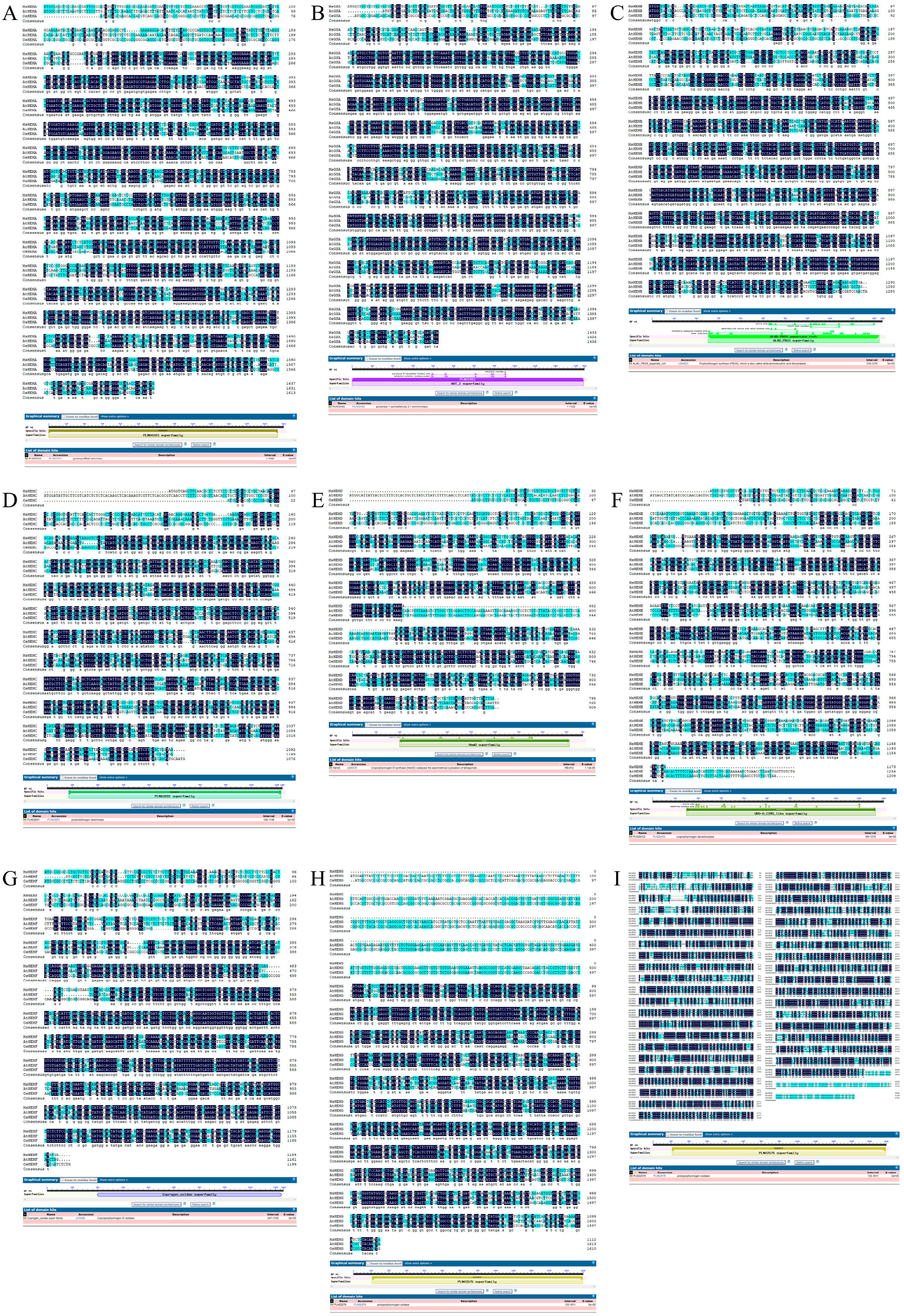
A
B
C
D
E
F
G
H
I

J

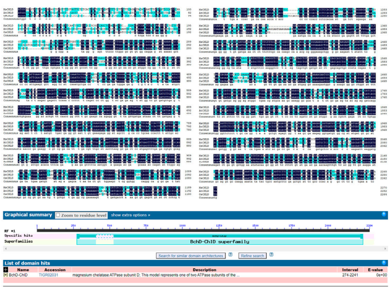

K

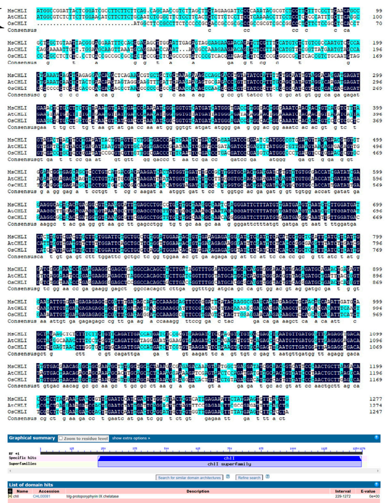

L

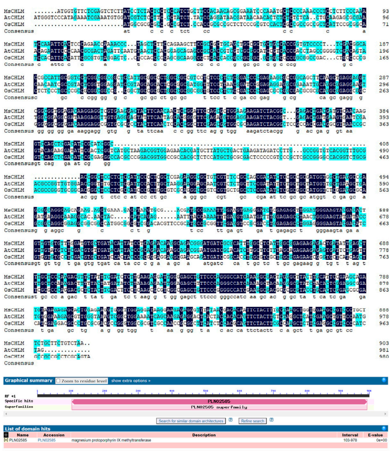

M

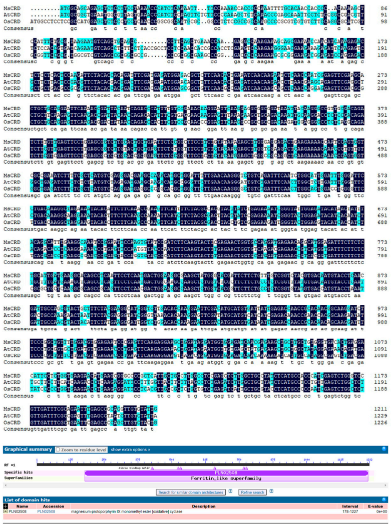

N

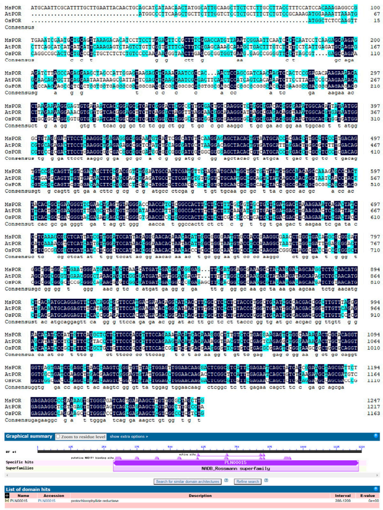

O

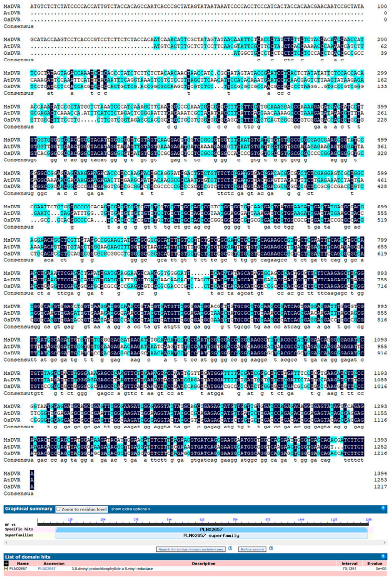

P

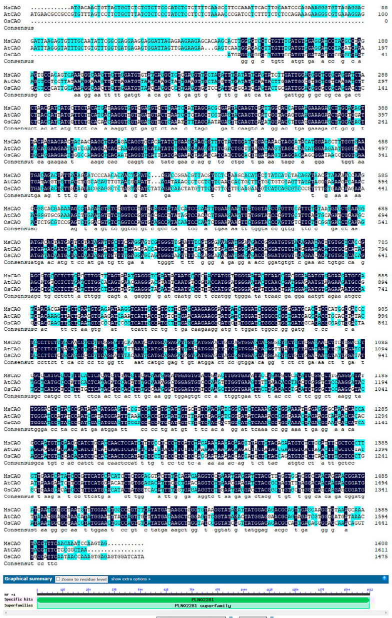

Q

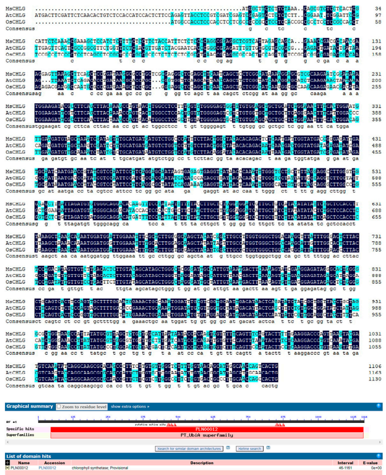

R

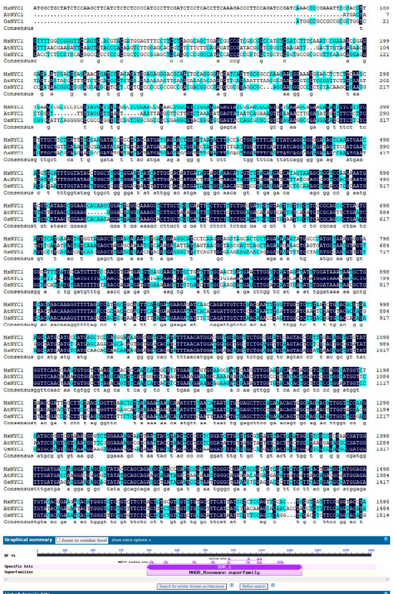

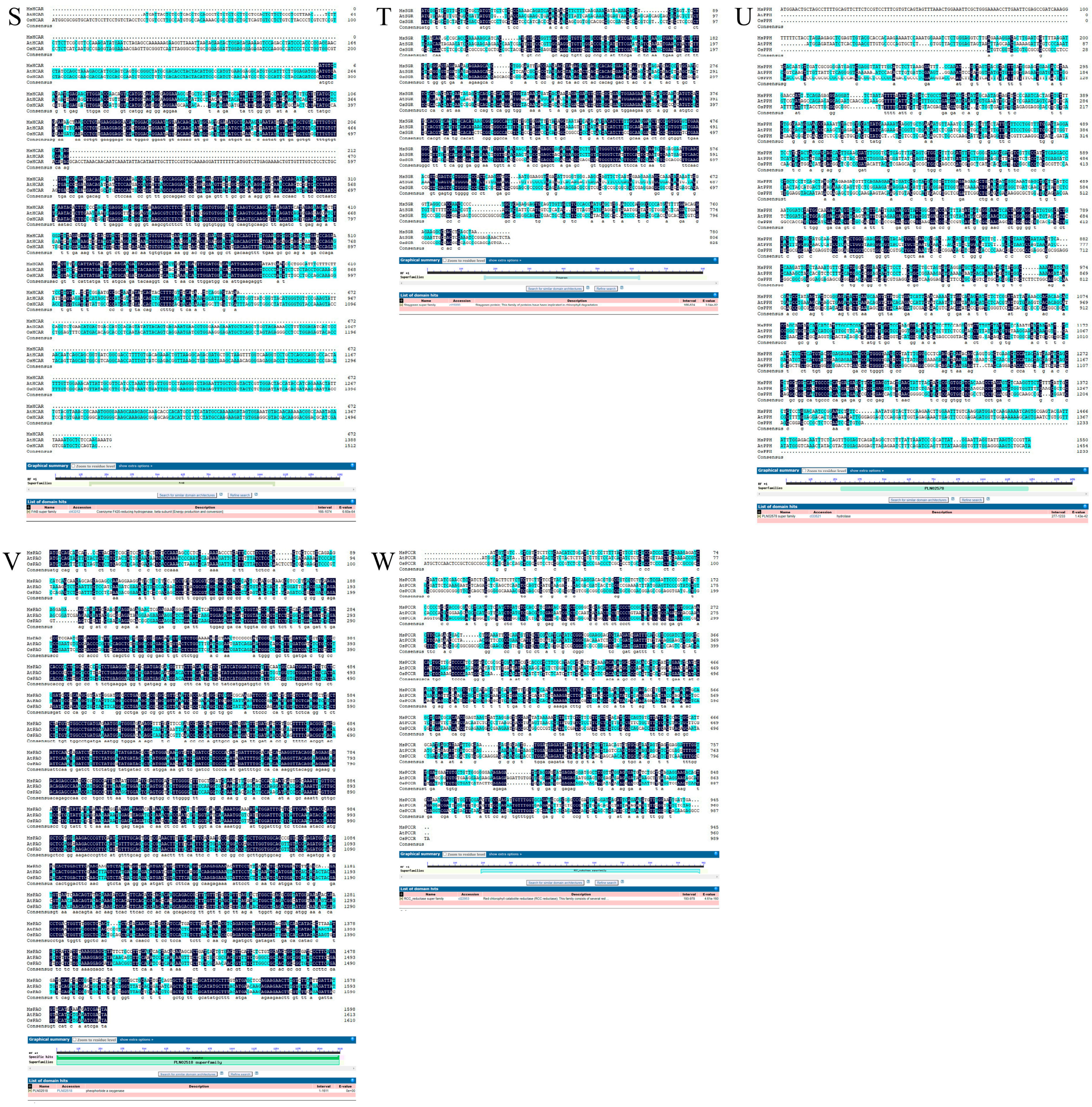

Figure S2. The multiple sequences alignment of chlorophyll biosynthesis and degradation genes between *M. sinostellata*, Arabidopsis and rice. A - W represent the sequences alignment of *HEMA*, *GSA*, *HEMB*, *HEMC*, *HEMD*, *HEME*, *HEMF*, *HEMG*, *CHLH*, *CHLD*, *CHLI*, *CHLM*, *CRD*, POR, *DVR*, *CAO*, *CHLG*, *NYC1*, *HCAR*, *SGR*, *PPH*, *PAO*, *PCCR*, respectively. The homologous sequences of each chlorophyll biosynthesis and degradation gene contain the same conserved domain.

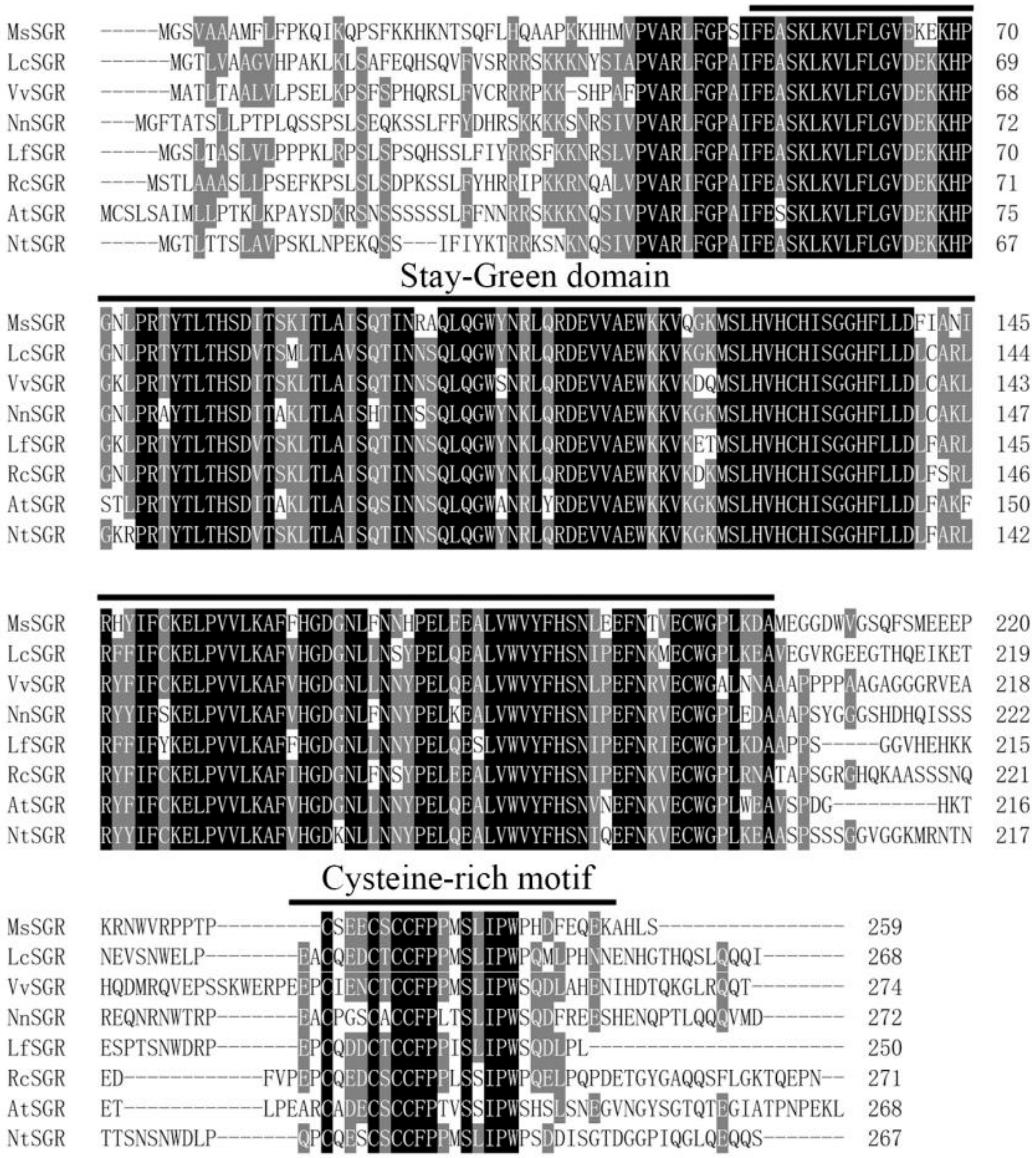

Figure S3. Multiple protein alignment of SGR homologs. The SGR homologs amino acid sequence used in the analysis are listed as follows: *Litchi chinensis* (AKA88530.1), *Vitis vinifer*a (XP_002282183.1), *Nelumbo nucifera* (XP_010255591.1), *Liquidambar formosana* (AQM49955.1), *Rosa chinensis* (XP_024170390.1), *Arabidopsis thaliana* (NP_001328989.1), *Nicotiana tabacum* (XP_016500144.1).

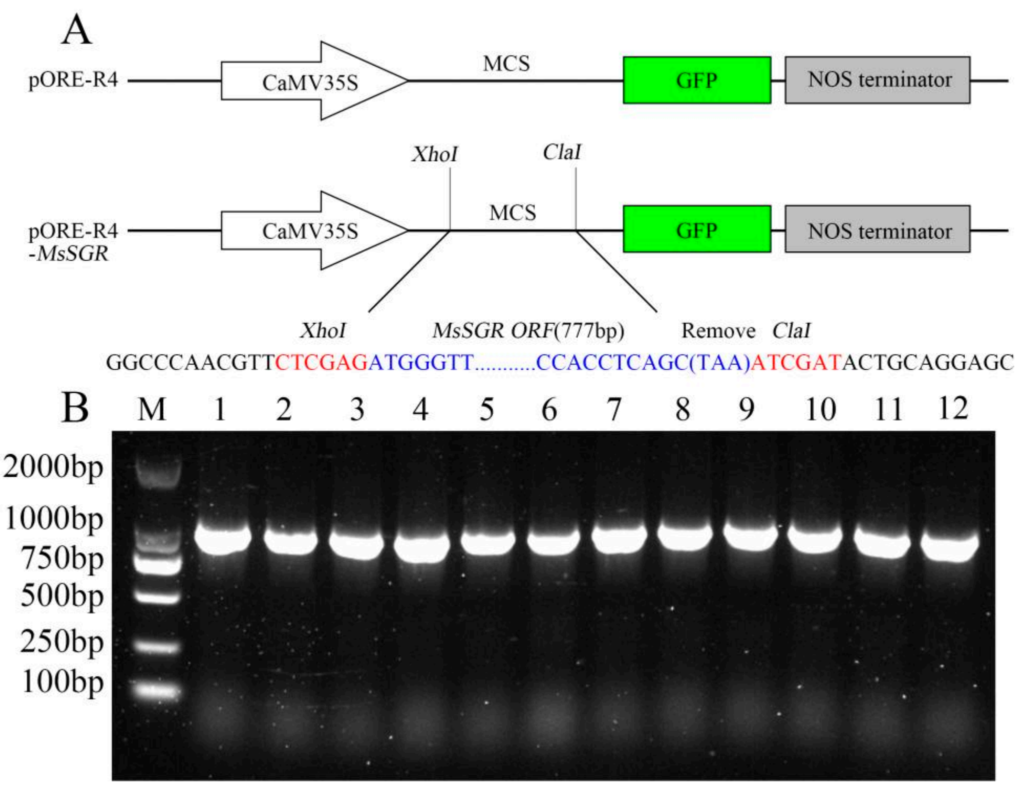

Figure S4. The Construction of plant expression vector. **(A)** Vector map of pORE-R4 and pORE-R4-*MsSGR*. **(B)** The PCR for colonies transformed with pORE-R4-*MsSGR.*

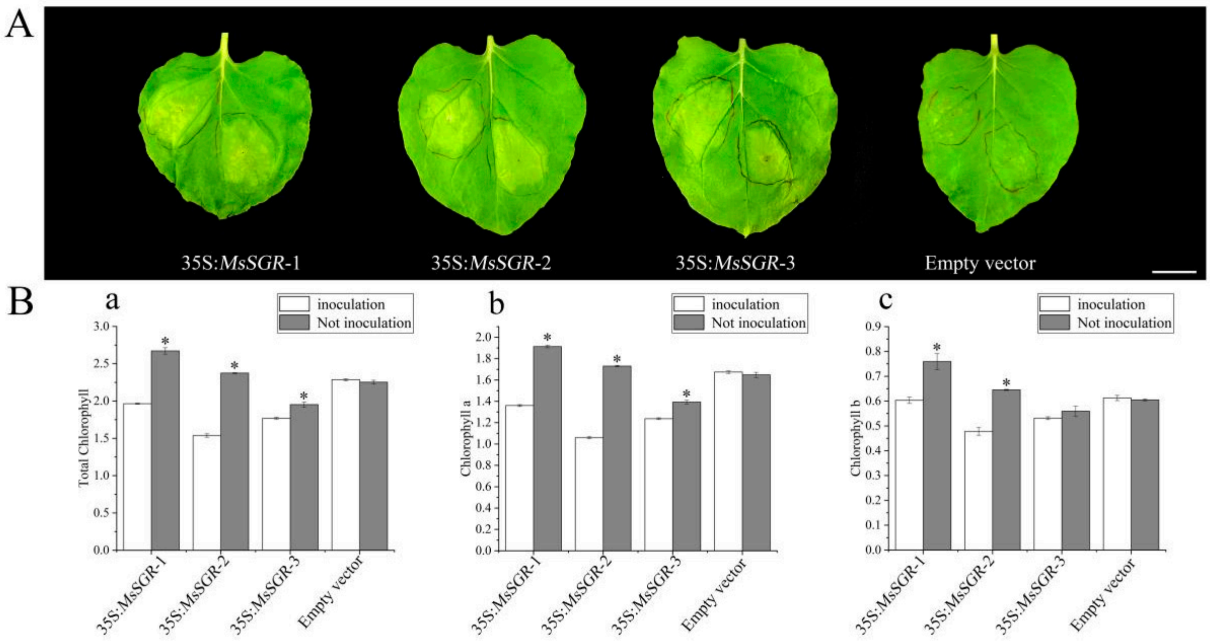

Figure S5. *MsSGR* promotes chlorophyll degradation in tobacco. **(A)** Transient overexpression of *MsSGR* in tobacco, bar=1 cm. **(B)** Chlorophyll contents of tobacco leaves inoculated by recombinant vectors transformed Agrobacterium and empty vectors transformed Agrobacterium. * represents significant difference(P<0.05).

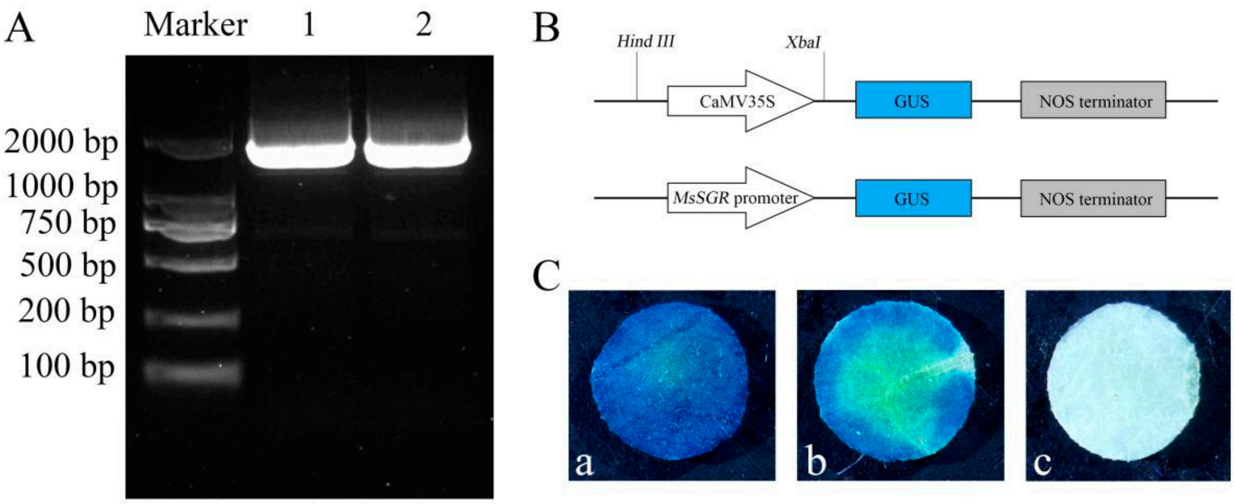

Figure S6. The cloning of *MsSGR* promoter and promoter activity assay. **(A)** Cloning of *MsSGR* promoter, 1-2 are two repetitions. **(B)** Vector map of PBI121-GUS and PIB121-MsSGR-GUS. **(C)** GUS enzyme activity analysis. a-c represents tobacco leaves inoculated by PBI121-GUS vector transformed Agrobacterium, PIB121-MsSGR-GUS vector transformed Agrobacterium and no vector transformed Agrobacterium.

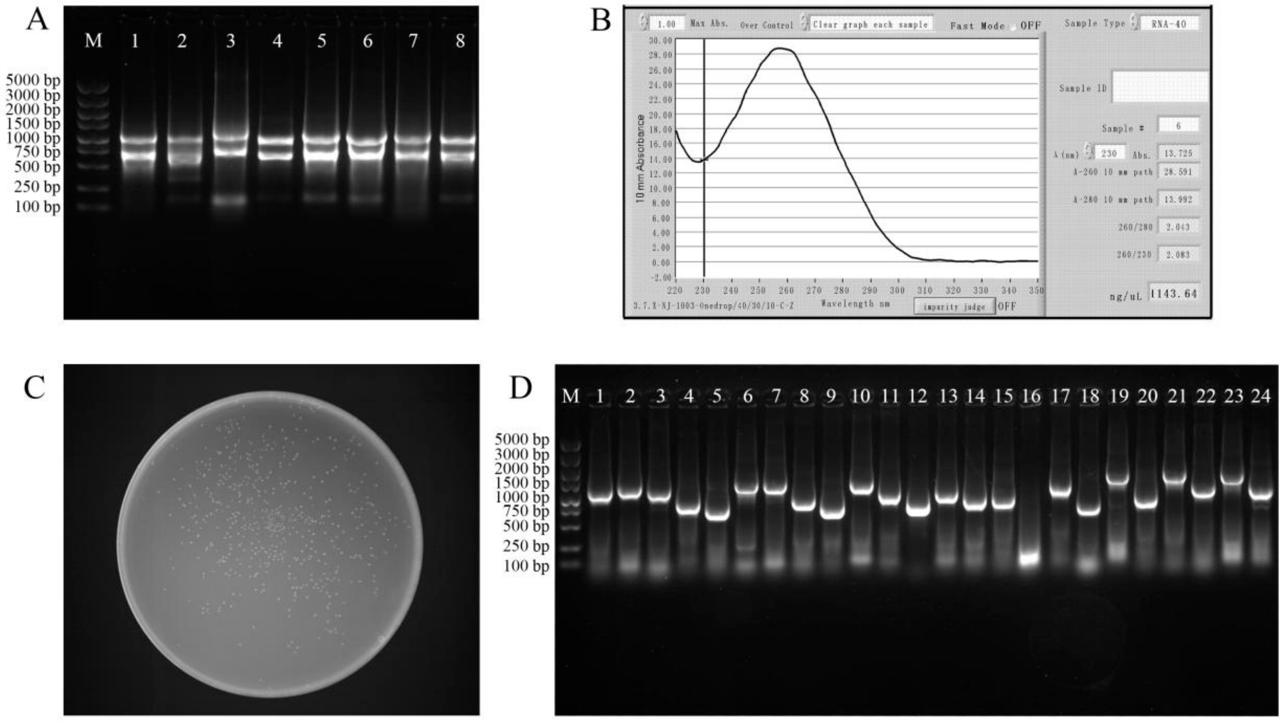

Figure S7 Construction of Yeast two-hybrid library of *M. sinostellata*. (A) Total RNA extraction from the leaves and leave buds of *M. sinostellata*. (B) Analysis of RNA concentration and purity. (C) Assessment of total library capacity. (D) Identification of insert sizes of Yeast two-hybrid library.

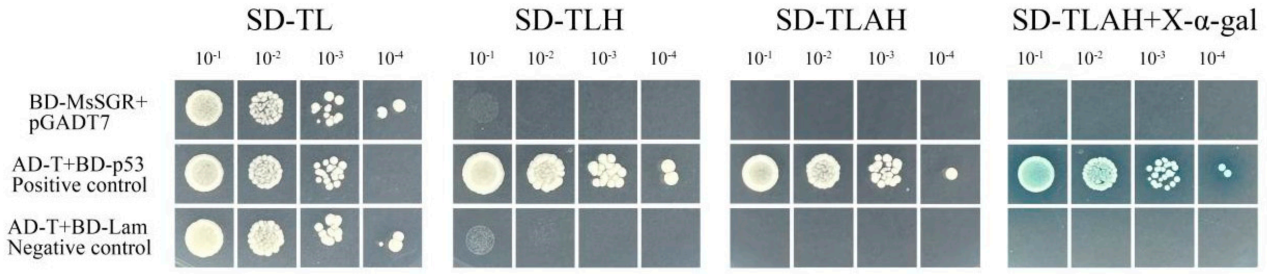

Figure S8 The self-activation and cell toxicity of MsSGR protein in yeast cells. Colonies grew on medium SD-Trp-Leu, SD-Trp-Leu-His, SD-Trp-Leu-His-Ade and SD-Trp-Leu-His-Ade+x-α-gal. "AD" represented for self-activation group (pGBKT7-MsSGR+pGADT7); "+" represented for positive control (pGBKT7-T+pGADT7-P53); "-" represented for negative control (pGBKT7-T+pGADT7-Lam). Yeast liquid dilution concentration: 10^{-1}, 10^{-2},10^{-3}, 10^{-4}.

TMHMM result

```
# MsSGR Length: 259
# MsSGR Number of predicted TMHs:  0
# MsSGR Exp number of AAs in TMHs: 0.01921
# MsSGR Exp number, first 60 AAs:  0.0013
# MsSGR Total prob of N-in:        0.01172
MsSGR   TMHMM2.0        outside      1   259
```

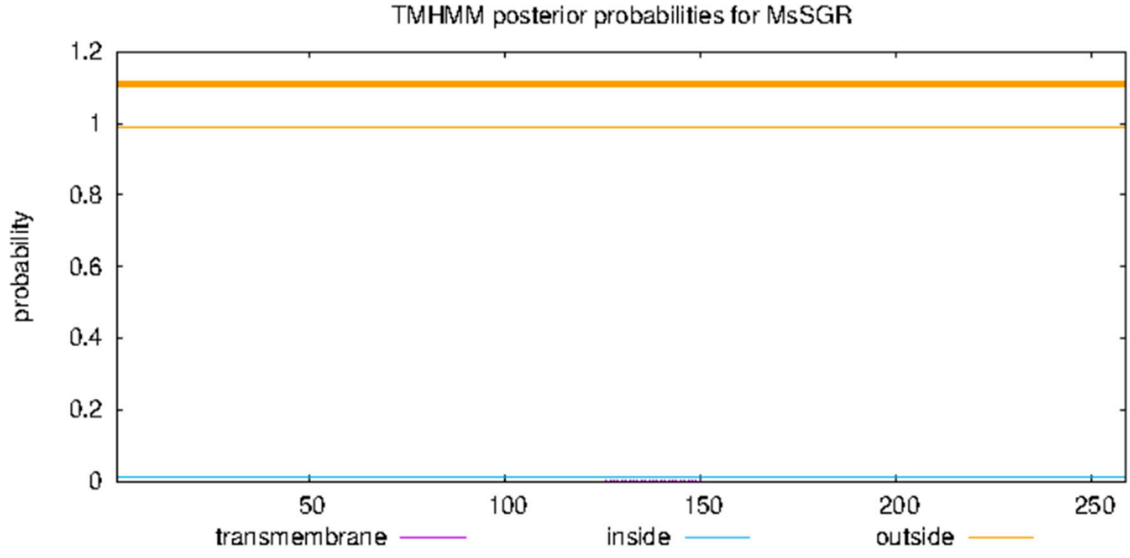

Figure S9 The transmembrane structure analysis of MsSGR protein.

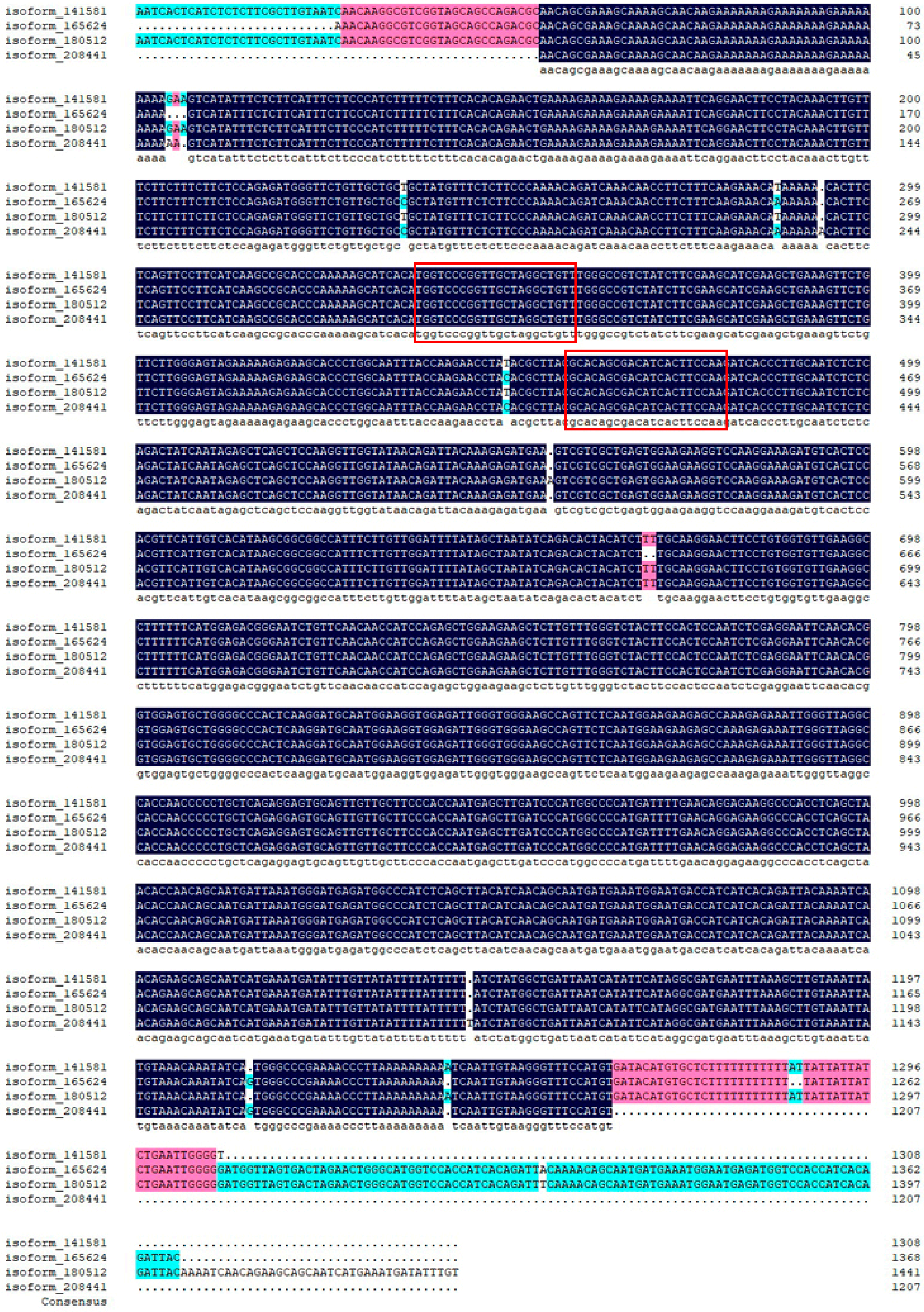

Figure S10 The primer design for qRT-PCR. The red boxes represent forward primer and reverse primer, respectively.

Table S1 The primer sequences. F represents the forward primer sequence; R represents the reverse primer sequence.

Primer name	Primer sequences (5'→3')	Function
MsSGR-F	AGATGGGTTCTGTTGCTGCTGCTAT	Gene cloning
MsSGR-R	TTGCTGTTGGTGTTAGCTGAGGTGG	
P-*MsSGR*-F	GTACGCAATCCACGTCCCTAAGCCT	Promoter cloning
P-*MsSGR*-R	AACATAGCAGCAGCAACAGAACCCA	
GUS-MsSGR-F	TGATTACGCCAAGCTTGTACGCAATCCACGTCCC	GUS analysis
GUS-MsSGR-R	CCGGGGATCCTCTAGACTCTGGAGAAGAAAGAAGAAACAAG	
MsSGR-R4-F	TTGGGGCCCAACGTTCTCGAGATGGGTTCTGTTGCTGCTGC	Subcellular localization and gene function
MsSGR-R4-R	TGGAGCTCCTGCAGTATCGATGCTGAGGTGGGCCTTCTCC	
MsSGR-BD-F	CATGGAGGCCGAATTCATGGGTTCTGTTGCTGCTGC	Yeast two-hybrid vector construction
MsSGR-BD-R	GGATCCCCGGGAATTCTTAGCTGAGGTGGGCCTTCTCC	
qPCR-*MsHEMA*-F	GATTGGAGCAGGCAAGATGG	qRT-PCR
qPCR-*MsHEMA*-R	CTTTCAGTTCCTCGCGGATG	
qPCR-*MsGSA*-F	TTGCACCATCGCAGTTTGAG	
qPCR-*MsGSA*-R	TATCAGCTGCAGCAATCGTC	
qPCR-*MsHEMB*-F	GTTGAAGAGGTTTACAAGGCACG	
qPCR-*MsHEMB*-R	TGACTTTAGAGCATCGGGAAT	
qPCR-*MsHEMC*-F	TATTGGTAGTGCTTCCCTCCG	
qPCR-*MsHEMC*-R	CGTGTCTGAACATTGCCTCTG	
qPCR-*MsHEMD*-F	CATACTCCACTGTACCTGCTCG	
qPCR-*MsHEMD*-R	CGGAATAAGATTAACCCAAGC	
qPCR-*MsHEME*-F	AACGGACGCATCCAGACCTAC	
qPCR-*MsHEME*-R	CGTCTCCTACCTTCCGCCATA	
qPCR-*MsHEMF*-F	GATAGAGCGACGGAAGGACAC	
qPCR-*MsHEMF*-R	TACCGCGATCATACACCAAGT	
qPCR-*MsHEMG*-F	TATGAAACGCCGGAAGGATTG	
qPCR-*MsHEMG*-R	CAGCAGCATTGGAAAGAGGAC	
qPCR-*MsCHLH*-F	CCGTGACGAATCGACTTACCA	
qPCR-*MsCHLH*-R	ATCCATCCTGTCTCGCTCCTT	
qPCR-*MsCHLD*-F	AGGATGTCAGTATCAGCAGGGAG	
qPCR-*MsCHLD*-R	AGCTAAGCATTTCGCAACTCG	
qPCR-*MsCHLI*-F	CACTCACAGAGGGCGTCAAGG	
qPCR-*MsCHLI*-R	ATTCCATCCAGAGGCAGCAGA	
qPCR-*MsCHLM*-F	TGCTCCCAAGACGTTCTATTTC	
qPCR-*MsCHLM*-R	TAAGCCCTAGTCGCCTTTGAT	
qPCR-*MsCRD*-F	AGGCGGCGGATAAATTACAAG	
qPCR-*MsCRD*-R	CAGTCTCCTCCCAAGCTCTTT	
qPCR-*MsPOR*-F	GATGGAGCGAAAGCCTACAAG	
qPCR-*MsPOR*-R	GTAGAGGGAGGCGAAGGTGAT	
qPCR-*MsDVR*-F	GTAGAGGGAGGCGAAGGTGAT	
qPCR-*MsDVR*-R	GCAGTTCTTGGAGGGTGTCTT	

qPCR-*MsCAO*-F	GTCCTGTCCAGCCATACCATC	
qPCR-*MsCAO*-R	TTCCCACGAAATACAACCCAC	
qPCR-*MsCHLG*-F	TATGAAACGCCGGAAGGATTG	
qPCR-*MsCHLG*-R	CAGCAGCATTGGAAAGAGGAC	
qPCR-*MsNYC1*-F	CATTATACGCAGCAGAGGCAGAC	
qPCR-*MsNYC1*-R	ACGACGCAGAGTGAGAAGACG	
qPCR-*MsHCAR*-F	CTGTAGAAGGAGCACAATGGA	
qPCR-*MsHCAR*-R	CTGGCTTTGGAGTAAGTCTGT	
qPCR-*MsSGR*-F	TGGTCCCGGTTGCTAGGCTGTT	
qPCR-*MsSGR*-R	TTGGAAGTGATGTCGCTGTGC	
qPCR-*MsPPH*-F	CCCCACCGTTGCTATTTCTTC	
qPCR-*MsPPH*-R	ACTCACGACCCAAATCCTTCA	
qPCR-*MsPAO*-F	CCCTTCTCCAACTCCTCCTTC	
qPCR-*MsPAO*-R	GCTTCTCCTTCTTCAGCGTCA	
qPCR-*MsPCCR*-F	CTGGACGCACAACGGAGTAAG	
qPCR-*MsPCCR*-R	CAATGGCAGTGGGAGATAAGA	
EF1-α-F	GATGATTCCAACCAAGCCCA	Reference gene
EF1-α-R	CACCCACTGCAACAGTCTGG	
